# Involvement of Proteasomal and Endoplasmic Reticulum Stress in Neurodegeneration After Global Brain Ischemia

**DOI:** 10.1007/s12035-023-03479-5

**Published:** 2023-07-14

**Authors:** Katarina Ziakova, Maria Kovalska, Ivana Pilchova, Katarina Dibdiakova, Maria Brodnanova, Michal Pokusa, Dagmar Kalenska, Peter Racay

**Affiliations:** 1 Biomedical Center, Jessenius Faculty of Medicine in Martin, Comenius University in Bratislava, Martin, Slovakia; 2 Department of Histology and Embryology, Jessenius Faculty of Medicine in Martin, Comenius University in Bratislava, Martin, Slovakia; 3 Department of Medical Biochemistry, Jessenius Faculty of Medicine in Martin, Comenius University in Bratislava, Mala Hora 4D, SK-03601 Martin, Slovak Republic; 4 Department of Anatomy, Jessenius Faculty of Medicine in Martin, Comenius University in Bratislava, Martin, Slovakia

**Keywords:** Global brain ischemia, Neurodegeneration, Ubiquitin proteasome system, Endoplasmic reticulum, Apoptosis

## Abstract

A brief period of transient global brain ischemia leads to selective ischemic neurodegeneration associated with death of hippocampal CA1 pyramidal neurons days after reperfusion. The mechanism of such selective and delayed neurodegeneration is still uncertain. Our work aimed to study the involvement of proteasomal and endoplasmic reticulum (ER) stress in ischemic neurodegeneration. We have performed laser scanning confocal microscopy analysis of brain slices from control and experimental animals that underwent global brain ischemia for 15 min and varying times of reperfusion. We have focused on ubiquitin, PUMA, a proapoptotic protein of the Bcl-2 family overexpressed in response to both proteasomal and ER stress, and p53, which controls expression of PUMA. We have also examined the expression of HRD1, an E3 ubiquitin ligase that was shown to be overexpressed after ER stress. We have also examined potential crosstalk between proteasomal and ER stress using cellular models of both proteasomal and ER stress. We demonstrate that global brain ischemia is associated with an appearance of distinct immunoreactivity of ubiquitin, PUMA and p53 in pyramidal neurons of the CA1 layer of the hippocampus 72 h after ischemic insults. Such changes correlate with a delay and selectivity of ischemic neurodegeneration. Immunoreactivity of HRD1 observed in all investigated regions of rat brain was transiently absent in both CA1 and CA3 pyramidal neurones 24 h after ischemia in the *hippocampus*, which does not correlate with a delay and selectivity of ischemic neurodegeneration. We do not document significant crosstalk between proteasomal and ER stress. Our results favour dysfunction of the ubiquitin proteasome system and consequent p53-induced expression of PUMA as the main mechanisms responsible for selective and delayed degeneration of pyramidal neurons of the hippocampal CA1 layer in response to global brain ischemia.

## Introduction

A brief period of transient global brain ischemia, arising in humans as a consequence of cardiac arrest or induced experimentally in animals, leads to selective ischemic neurodegeneration associated with the death of hippocampal CA1 pyramidal neurons days after reperfusion in humans [1–3] and rodents [4, 5]. Other neurons, e.g., hippocampal CA3 pyramidal neurons or parietal cortical pyramidal neurons, are much less vulnerable [4]. Prolonged ischemia is further associated with delayed death of neurons of caudate and thalamic nuclei [4, 6, 7] as well as the death of Purkinje neurons of the cerebellar cortex [1, 3, 8]. Depending on the duration of initial ischemia, death of hippocampal CA1 pyramidal neurons usually occurs 2–4 days after the initial ischemic insult. Therefore, this phenomenon is commonly referred to as delayed neuronal death [9]. Since ischemic neurodegeneration results in severe neurologic and cognitive deficits in persons that survived cardiopulmonary resuscitation after cardiac arrest [10, 11], the mechanism of ischemic neurodegeneration associated with delayed neuronal death was extensively studied for several years. Previous hypotheses of selective and delayed ischemic neurodegeneration were based on glutamate excitotoxicity [12, 13], irreversible inhibition of protein synthesis [14, 15], excessive degradation of proteins [16], mitochondrial dysfunction [17–19], the stress of endoplasmic reticulum [20–22] (ER) and mitochondrial apoptosis [23]. All previous hypotheses were based on experimental findings, but could not explain selectivity and delay of post-ischemic neuronal death.

Dysfunction of the ubiquitin proteasome system (UPS) specific to the hippocampal CA1 region has been documented after transient global brain ischemia in previous studies [24, 25]. Further experiments also supported the involvement of UPS dysfunction in selective and delayed neurodegeneration after transient global brain ischemia. Selective degeneration of CA1 hippocampal neurons was also observed after stereotactic microinjection of proteasome inhibitor epoxomicin into mouse hippocampus [26]. Similarly, CA1 pyramidal neurons, but not neurons within the CA3 or dentate gyrus (DG) regions, died after incubation of organotypic hippocampal slice cultures (OHSCs) with epoxomicin [27]. Delayed neuronal death after transient global brain ischemia has also been attributed to the accumulation of ubiquitin-conjugated protein aggregates [28]. Concerning the cause and the consequence of the post-ischemic accumulation of ubiquitin-conjugated protein aggregates, conflicting data have been reported [29, 30]. Increased levels of ubiquitin-conjugated proteins were detected throughout all hippocampus regions while only CA1 pyramidal neurons were affected after incubation of OHSCs with epoxomicin [27]. The results of our previous study indicated that post-ischemic inhibition of 26 S proteasome is a cause of the accumulation of ubiquitin-conjugated protein aggregates after global brain ischemia [31]. Our further study did not support direct cytotoxicity of the aggregates of ubiquitin-conjugated proteins [32] but indicated that proteasome inhibition leads to both transcription-dependent and -independent changes in the expression of pro-apoptotic proteins, PUMA and Noxa, resulting in the initiation of mitochondrial apoptosis through caspase 3 activation [32].

UPS is also closely associated with the stress of ER that can be induced by different extracellular and intracellular conditions, including ischemia [33]. In response to ER stress, cells trigger an adaptive signalling pathway called the unfolded protein response (UPR). Depending on the disturbing agent/condition and/or intensity/duration of the ER stress, UPR can help cells to cope with the stress by attenuation of protein synthesis, degradation of the unfolded/misfolded proteins, and increase of the capacity of the ER to fold proteins [34]. Removal of unfolded/misfolded proteins from the lumen of ER involves a cellular pathway called ER-associated degradation (ERAD). In the process of ERAD, aberrant proteins are retro-translocated from ER to cytoplasm, ubiquitinated and degraded by 26 S proteasome [35, 36]. Prolonged or too intensive activation of the UPR results in cellular switches from pro-survival to pro-death response that leads to different forms of cell death, including mitochondrial apoptosis [33, 37]. Pro-apoptotic proteins PUMA, Noxa and BIM seem to be pivotal components of ER stress-induced mitochondrial apoptosis [37].

Several previous studies have investigated the involvement of both proteasomal and ER stress in the mechanism of neurodegeneration after global brain ischemia. But a limited number of studies focused on simultaneous investigations of ischemia-induced proteasome and ER stress as the mechanisms of selective and delayed degeneration of pyramidal neurons of the hippocampal CA1 layer in response to global brain ischemia. In this study, we have performed laser scanning confocal microscopy (LSCM) analysis of brain slices from control and experimental animals, which underwent global brain ischemia in 15 min and different times of reperfusion, with a focus on spatio-temporal changes of expression of ubiquitin, PUMA, p53 and HRD1. PUMA, a pro-apoptotic protein of the Bcl-2 family [38], was suggested to be a key mediator of selective death of hippocampal CA1 neurons after proteasomal stress [26, 27]. The expression of PUMA is under the transcriptional control of p53 [38]. HRD1 is an E3 ubiquitin ligase that plays a central role of ERAD activation in response to ER stress [33, 36]. It is involved in the ubiquitination and retro-translocation of aberrant proteins from ER to the cytoplasm for 26 S proteasome degradation [39–41]. It was previously shown that increased expression of HRD1 represents another typical response of neuronal cells to ER stress [42]. In addition, we have examined possible crosstalk between proteasomal and ER stress by analysis of the expression of key proteins of both stresses (HRD1 as representative of ER stress and HSP70 for proteasomal stress) in their cellular models using neuroblastoma SH-SY5Y cells.

## Materials and Methods

### Ischemia-Reperfusion

Animal studies were carried out in line with the principles of the Declaration of Helsinki and according to the guideline for Animal Care and Health of the State Veterinary and Food Department of the Slovak Republic, as well as ARRIVE guidelines [43]. Experiments were approved by the ethics committee of Jessenius Faculty of Medicine in Martin, Comenius University in Bratislava (approval number EK 48/2019) and by the State Veterinary and Food Department of the Slovak Republic (approval number Ro-1360/2020 − 220). Experiments were implemented in accordance with Directive 2010/63/EU of the European Parliament and the Council for the protection of animals used for scientific purposes.

A total of 50 adult male Wistar rats (Velaz, Prague, Czech Republic) were used. All animals were maintained on a 12/12-hour light/dark cycle. Food and water were available ad libitum until the beginning of the experiments. Animal health and behaviour were monitored regularly by a doctor of veterinary medicine. Transient global cerebral ischemia was produced using the four-vessel occlusion model described previously [44]. Briefly, on day 1, both vertebral arteries were irreversibly occluded by coagulation through the alar foramina after anaesthesia with a mixture of 2% halothane, 30% O_2_, and 68% N_2_O. On day 2, both common carotid arteries were occluded for 15 min by small clips under anaesthesia with a mixture of 2% halothane, 30% O_2_, and 68% N_2_O. Two minutes before carotid occlusion, the halothane was removed from the mixture. Body temperature was maintained by means of a homeothermic blanket. Global ischemia was followed by 24 or 72 h of reperfusion induced by release of carotid occlusion. Control animals underwent the same procedure except for carotid occlusion. Immediately after the treatment, control and experimental animals were anaesthetised, perfused transcardially with ice-cold 0.1 mol/l phosphate-buffered saline (PBS, pH 7.4) and fixed by perfusion with ice-cold 4% paraformaldehyde in PBS. The brains were removed, postfixed with the same solution as above for 24 h at 4 °C, and cryoprotected by infiltration using 30% sucrose for the next 24 h at 4 °C.

The rats were randomised into the following experimental groups: (i) Control sham‑operated rats; (ii) rats that underwent a 15‑min global brain ischemia followed by 24 h of reperfusion (I24R; and iii) rats that underwent a 15‑min global brain ischemia followed by 72 h of reperfusion (I72R). Ischemic animals were included in the study if they underwent successful occlusion of both common carotid arteries defined by bilateral dilation of pupils and lack of pupillary light reflex.

### Detection of Ubiquitin, PUMA, p53 and HRD1

Ubiquitin, PUMA, p53 and HRD1 were detected in brain slices of control and experimental rats by immunofluorescence using laser scanning confocal microscopy as described previously [44]. The brains from control and experimental rats (without performing any blinding procedure) were frozen and sectioned with a cryostat at a thickness of 30 μm, and the sections were mounted onto Superfrost Plus glass (Thermo scientific). Mounted brain sections were permeabilised with a permeabilisation solution (0.1% Triton X-100 with 10% BSA) for 1 h. Mouse monoclonal antibodies against ubiquitin (1:50; SC-8017, Santa Cruz Biotechnology) and p53 (1:50, SC-55476, Santa Cruz Biotechnology) as well as rabbit polyclonal antibodies against HRD1 (1:50, 13473-1-AP, Proteintech) and PUMA (1:50, SC-28226, Santa Cruz Biotechnology) were used as primary antibodies. Tissue sections were incubated overnight at 4 °C in primary antibodies diluted in permeabilisation solution. Alexa Fluor 488 goat-anti-rabbit IgG (1:100, #4412, Cell Signaling Technology) was applied as a secondary antibody for HRD1 and PUMA, and Alexa Fluor 488 goat-anti-mouse IgG (1:100, A11001, Life Technologies) was used as a secondary antibody for ubiquitin and p53. Finally, the brain sections were cover-slipped with Fluoromount-G medium with addition of 4′,6-diamidino-2-phenylindole (DAPI, CA 0100 − 20, SouthernBiotech). In the absence of a primary antibody, no immunoreactivity was observed. The slides were examined by an Olympus FluoView FV10i confocal laser scanning microscope (Olympus) equipped with an objective of 10× with zoom up to 40x magnification and filters for FITC (fluorescein isothiocyanate for Alexa Fluor 488, excitation: 499 nm; emission: 520 nm) and Texas Red (excitation: 590 nm; emission: 618 nm)). Image capture was performed with Olympus Fluoview FV10-ASW software, version 02.01 (Olympus) and Quick Photo Micro software, version 2.3 (Promicra) and further processed in Adobe Photoshop CS3 Extended, version 10.0 for Windows (Adobe Systems, San Jose, CA, USA).

The brightness and contrast of each image file were uniformly calibrated by using Adobe Photoshop CS3 Extended, version 10.0 for Windows (Adobe Systems). Values of background staining were obtained and subtracted from the immunoreactive intensities.

### Fluoro-Jade C Staining as a Marker of Neuronal Degeneration

Neurodegeneration was monitored by Fluoro-Jade C staining of brain slices of control and experimental rats, followed by laser scanning confocal microscopy as described previously [45]. Brain slices from control rats and rats that underwent 15-minute global brain ischemia followed by 24 and 72 h of reperfusion were mounted on the Superfrost Plus glass (Thermo scientific) and dried for 30 min at 55 °C. The slices were then rinsed in descending grades of alcohol (absolute alcohol for 3 min, then 1 min in 70% alcohol) and 1 min in distilled water. After rehydratation, slices were incubated in 0.06% potassium permanganate solution for 15 min and rinsing for 2 min in redistilled water. The slices were then placed in 0.0001% Fluoro-Jade C (Millipore) staining solution for 2 h, followed by washing for 3 × 1 min in redistilled water. The slices were air-dried and cover-slipped with Fluoromount Aqueous Mounting Medium (F4680, Sigma-Aldrich) according to standard protocols.

### Cell Culture and Treatment

Neuroblastoma SH-SY5Y cells (ATCC) were cultivated and treated as described previously [42]. SH-SY5Y cells were maintained in DMEM:F12 (1:1) medium supplemented with 10% fetal bovine serum, 1% penicillin-streptomycin (PAA) at an optimal cell density of 0.5 × 10^6^ cells/mL at 37 °C and under a 5% CO_2_ humidified atmosphere. The media were changed every 3 days.

SH-SY5Y cells were treated with the indicated concentrations of tunicamycin, thapsigargin and bortezomib for indicated time intervals at 37 °C and under a 5% CO_2_ humidified atmosphere. At the end of the cultivation of control cells and the treatment of treated cells, the cells were washed 3 times with ice-cold phosphate-buffered saline (PBS) and then re-suspended in a lysis buffer (30 mmol/L Tris-HCl, 150 mmol/L NaCl, 1% CHAPS, 1x protease inhibitor cocktail, pH = 7.6) for total protein extraction. Protein concentrations were determined by a protein DC assay kit (Bio-Rad) with BSA as a standard.

### Western Blot Analysis

Western blot analysis of the expression of HRD1, HSP70 and ubiquitin in lysates of control or treated SH-SY5Y cells was performed as described previously [42].

Isolated proteins (30 µg per lane) were separated by sodium dodecyl sulphate-polyacrylamide gel electrophoresis (SDS-PAGE). After electrophoresis, separated proteins were transferred onto nitrocellulose membranes using a semi-dry transfer protocol. The membranes were controlled for even load and possible transfer artefacts by staining with Ponceau Red solution. After being blocked with BSA blocking buffer (50 mM Tris-Cl, pH 7.5, 150 mM NaCl, 0.05% Tween 20, 2% BSA), membranes were first incubated for 90 min with primary mouse monoclonal antibody against ubiquitin (1:1000; SC-8017, Santa Cruz Biotechnology), HSP70 (1:1000; SC-66048, Santa Cruz Biotechnology), and β-actin (1:2000, SC-47778, Santa Cruz Biotechnology) and rabbit polyclonal antibodies against HRD1 (1:1000, 13473-1-AP, Proteintech) dissolved in BSA blocking solution. Membranes incubated with primary antibodies were washed in TBS-T solution (50 mM Tris-Cl, pH 7.5, 150 mM NaCl, 0.05% Tween 20) and then incubated with secondary antibodies conjugated with horseradish peroxidase (1:5000, Santa Cruz). After extensive washes with TBS-T solution (4 times, 15 min), membranes were incubated in SuperSignal West Pico Chemiluminescent Substrate (Thermo scientific) solution for 3 min. Following exposure of the membranes to Chemidoc XRS (BioRad), the bands of the corresponding proteins were visualised by Quantity One software (BioRad).

## Results

**Fig. 1:**
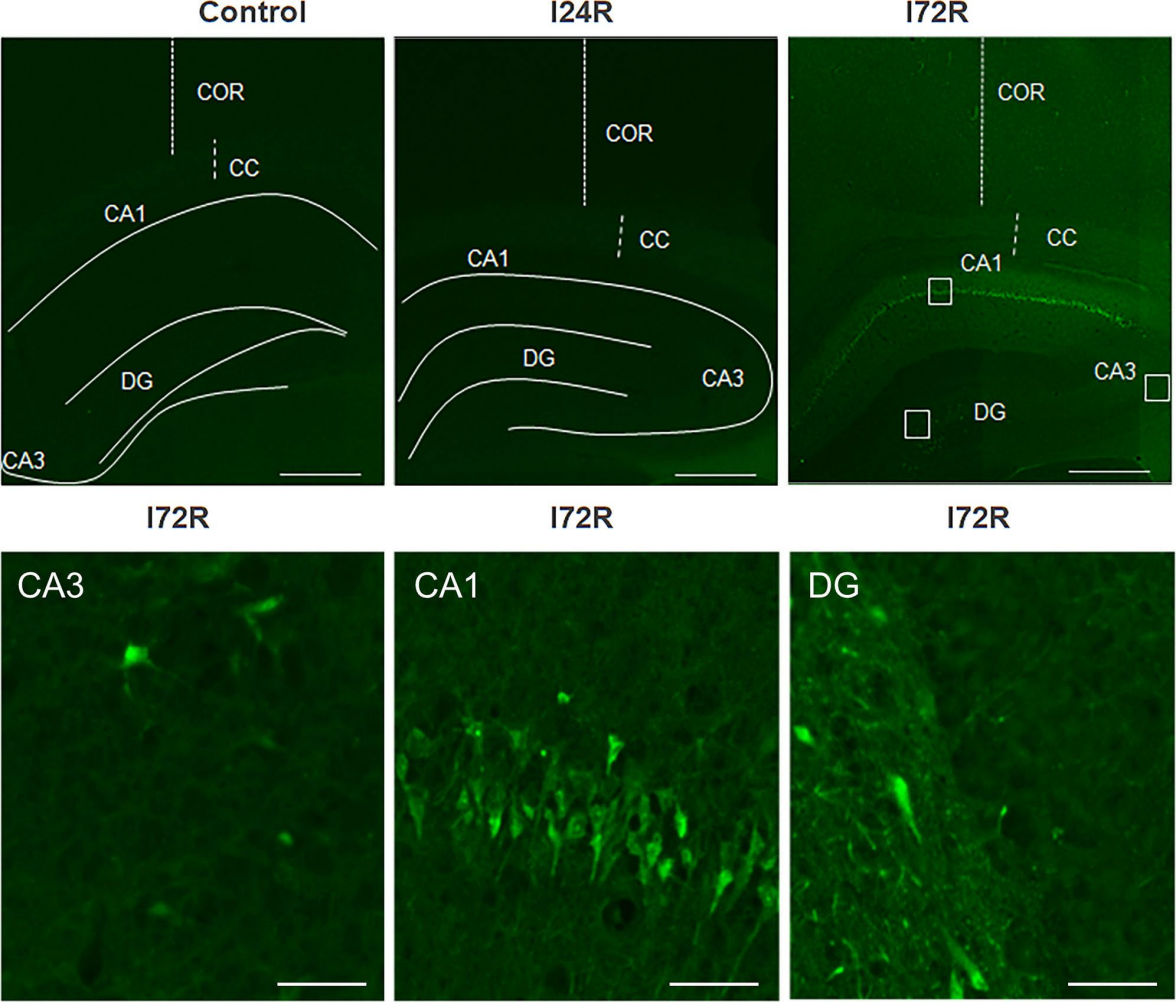
Effect of ischemia/reperfusion on cell degeneration. Fluorescent micrographs of Fluoro-Jade C staining of brain slices of control rats and rats that underwent 15 min global brain ischemia followed with reperfusion in duration 24 (I24R) and 72 h (I72R) with details of corresponding groups focusing on CA3, CA1 and DG regions of hippocampus of rats that underwent 15 min global brain ischemia followed with reperfusion in duration 72 h. White squares in the I72R figure represent area of magnification. Bar = 500 μm (upper figures); Bar = 50 μm (lower figures)

We have first examined the effect of global brain ischemia on neuronal degeneration using Fluoro-Jade C staining that results in contrasting and high-resolution labelling of degenerating neurones regardless of specific insult or mechanism of cell death [46]. We have not observed any Fluoro-Jade C positivity in control brains and brains from rats that underwent global brain ischemia followed by 24 h of reperfusion (Fig. 4). Fluoro-Jade C positivity was detected predominantly in the CA1 region 72 h after ischemia/reperfusion (Fig. 1). At the same time, a few Fluoro-Jade C positive cells were also observed in the CA3 and DG regions of the hippocampus (Fig. 1).

**Fig. 2:**
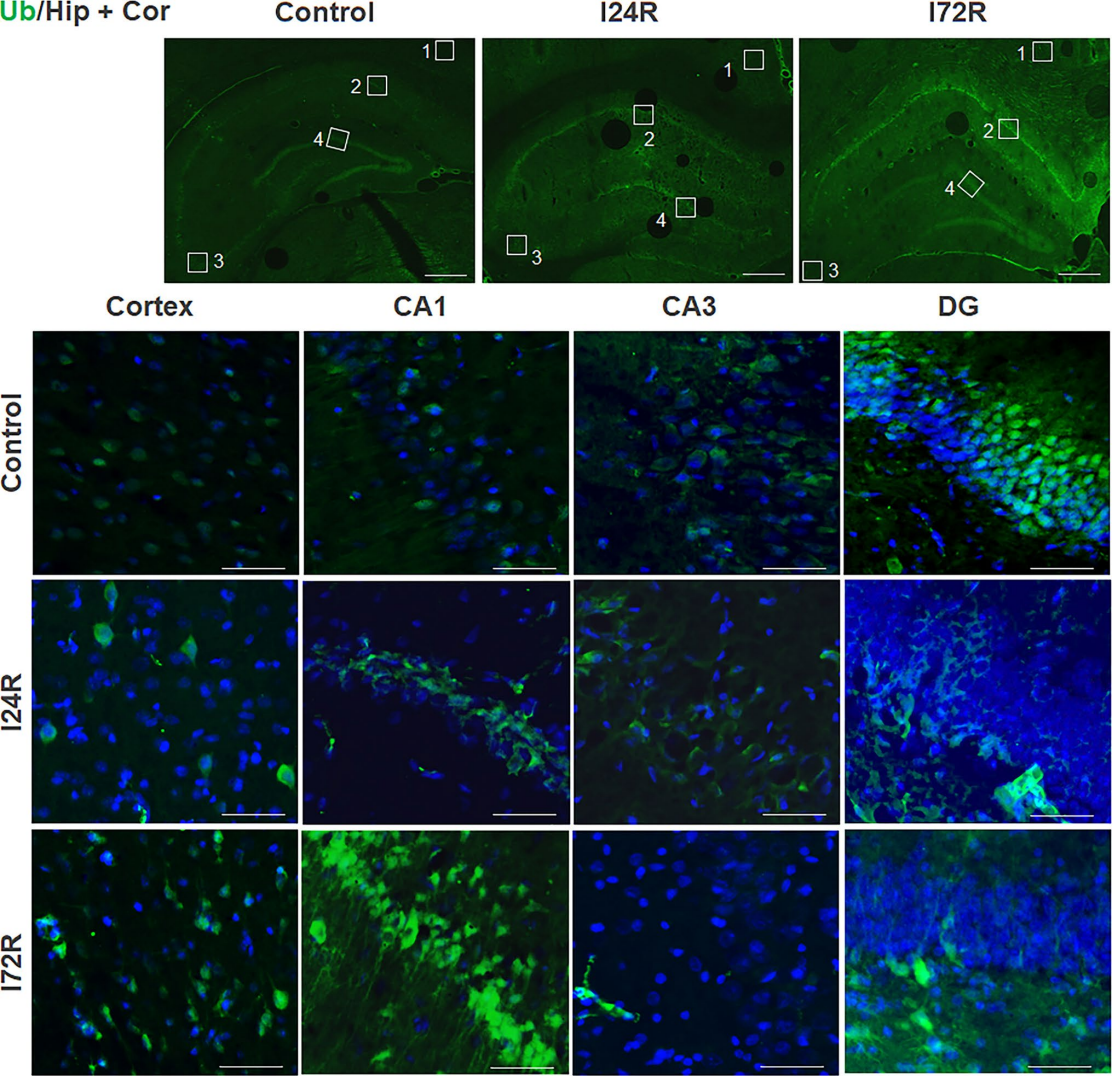
Effect of ischemia/reperfusion on ubiquitin immunoreactivity. Fluorescence micrographs of cells positive for ubiquitin (green) of brain slices of control rats and rats that underwent 15 min global brain ischemia followed with reperfusion in duration 24 (I24R) and 72 h (I72R) with details of corresponding groups focusing on CA3, CA1 and DG regions of hippocampus and cortex. Nuclei are co-stained with DAPI (blue). White squares in the low magnification figures represent area of magnification; cortex (1), CA1 (2), CA3 (3) and DG (4). Bar = 500 μm (upper figures); Bar = 50 μm (the rest of figures)

A faint ubiquitin staining, without distinct morphological features, was detected in the neocortex and the hippocampal CA1 and CA3. Ubiquitin immunoreactive neurones were observed in DG region of control brains (Fig. 2). At 24 h after ischemia, moderate ubiquitin immunoreactivity was observed in some neurons of neocortex, CA1 and CA3 pyramidal neurons (Fig. 2) and in some neuronal cells of DG polymorphic layer (Fig. 2). At 72 h after ischemia, distinct ubiquitin immunoreactivity was observed in CA1 pyramidal neurons (Fig. 2). In addition, ubiquitin immunoreactivity remained moderate in some neurons of neocortex and some neuronal cells of DG polymorphic layer (Fig. 2).

**Fig. 3:**
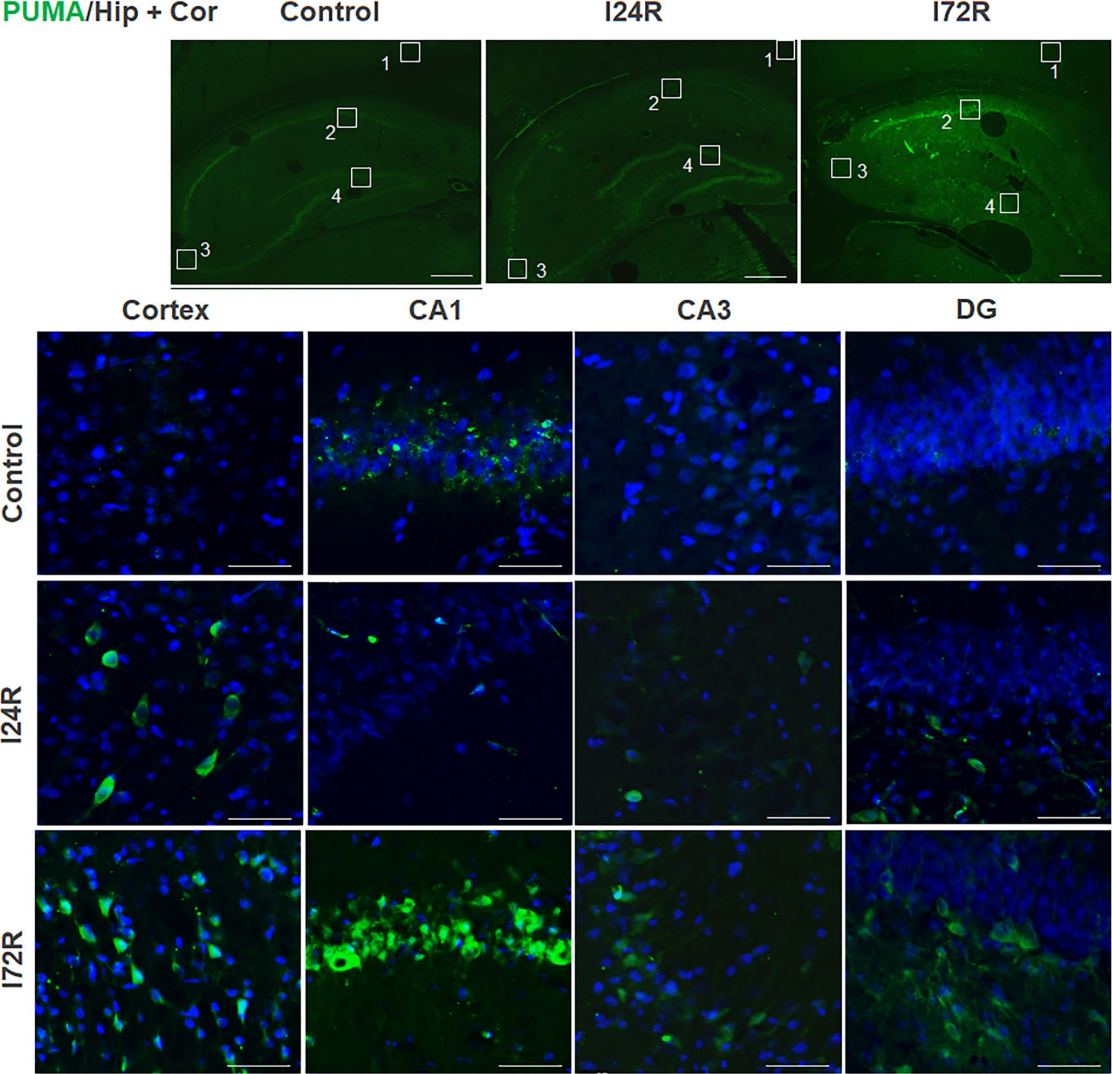
Effect of ischemia/reperfusion on PUMA immunoreactivity. Fluorescence micrographs of cells positive for PUMA (green) of brain slices of control rats and rats that underwent 15 min global brain ischemia followed with reperfusion in duration 24 (I24R) and 72 h (I72R) with details of corresponding groups focusing on CA3, CA1 and DG regions of hippocampus and cortex. Nuclei are co-stained with DAPI (blue). White squares in the low magnification figures represent area of magnification; cortex (1), CA1 (2), CA3 (3) and DG (4). Bar = 500 μm (upper figures ); Bar = 50 μm (the rest of figures)

A weak PUMA staining, without distinct morphological features, was detected predominantly in the hippocampal CA1 region of control brains (Fig. 3). At 24 h after ischemia, moderate PUMA immunoreactivity was observed mainly in some neurons of the neocortex and some neuronal cells of CA3 and DG polymorphic layer (Fig. 3). At 72 h after ischemia, distinct PUMA immunoreactivity was observed in CA1 pyramidal neurons (Fig. 3). In addition, moderate PUMA immunoreactivity was observed in some neurons of neocortex, CA3 pyramidal neurons (Fig. 3) and in some neuronal cells of DG polymorphic layer (Fig. 3).

**Fig. 4:**
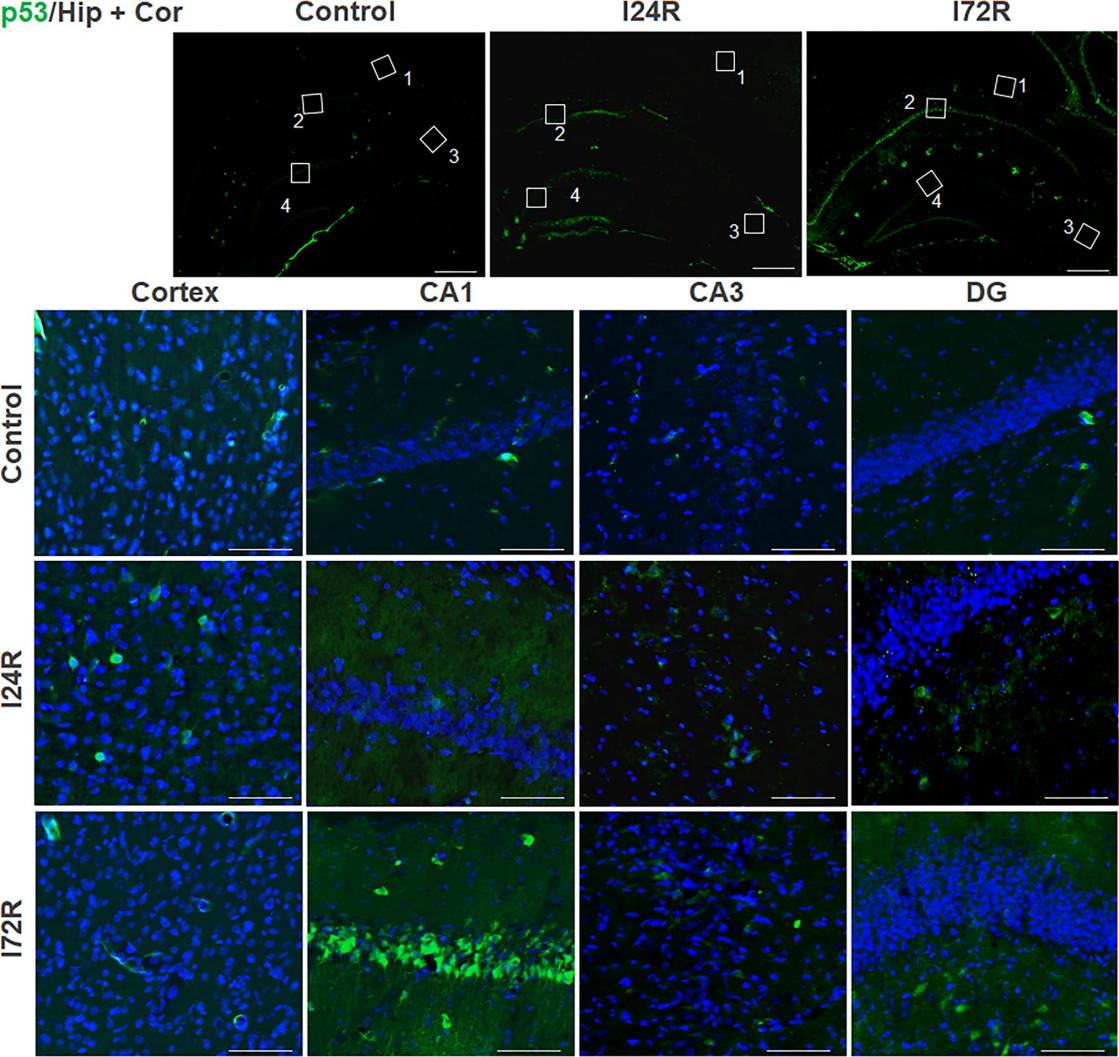
Effect of ischemia/reperfusion on p53 immunoreactivity.  Fluorescence micrographs of cells positive for p53 (green) of brain slices of control rats and rats that underwent 15 min global brain ischemia followed with reperfusion in duration 24 (I24R) and 72 h (I72R) with details of corresponding groups focusing on CA3, CA1 and DG regions of hippocampus and cortex. Nuclei are co-stained with DAPI (blue). White squares in the low magnification figures represent area of magnification; cortex (1), CA1 (2), CA3 (3) and DG (4). Bar = 500 μm (upper figures); Bar = 50 μm (the rest of figures)

A weak p53 staining, without distinct morphological features, was detected in the brains of control rats and brains of rats that underwent global brain ischemia for 15 min followed by 24 h of reperfusion (Fig. 4). At 72 h after ischemia, distinct p53 immunoreactivity was observed in CA1 pyramidal neurons (Fig. 4). In addition, moderate p53 was observed outside of DG polymorphic layer (Fig. 4).

**Fig. 5:**
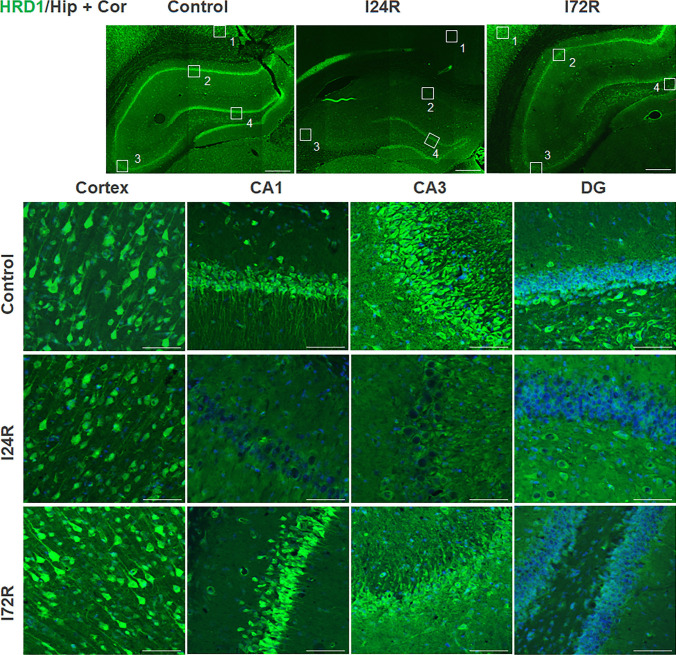
Effect of ischemia/reperfusion on HRD1 immunoreactivity. Fluorescence micrographs of cells positive for HRD1 (green) of brain slices of control rats and rats that underwent 15 min global brain ischemia followed with reperfusion in duration 24 (I24R) and 72 h (I72R) with details of corresponding groups focusing on CA3, CA1 and DG regions of hippocampus and cortex. Nuclei are co-stained with DAPI (blue). White squares in the low magnification figures represent area of magnification; cortex (1), CA1 (2), CA3 (3) and DG (4). Bar = 500 μm (upper figures); Bar = 50 μm (the rest of figures)

Distinct HRD1 immunoreactivity was present in neuronal cell bodies and processes throughout the brains of control rats (Fig. 5). Staining for HRD1 was detected in neurons of the neocortex, pyramidal neurons in the hippocampal CAl and CA3 layers and neurons of DG polymorphic layer. At 24 h after ischemia, distinct HRD1 immunoreactivity was still present in neurons of the neocortex, while HRD1 immunoreactivity almost disappeared in pyramidal neurons of both CA1 layer, representing vulnerable neurons, and CA3 layer, representing resistant neurons (Fig. 5) and in some neuronal cells of the DG polymorphic layer and (Fig. 5). Distinct HRD1 immunoreactivity was observed in all investigated brain regions 72 h after ischemia (Fig. 5).

**Fig. 6:**
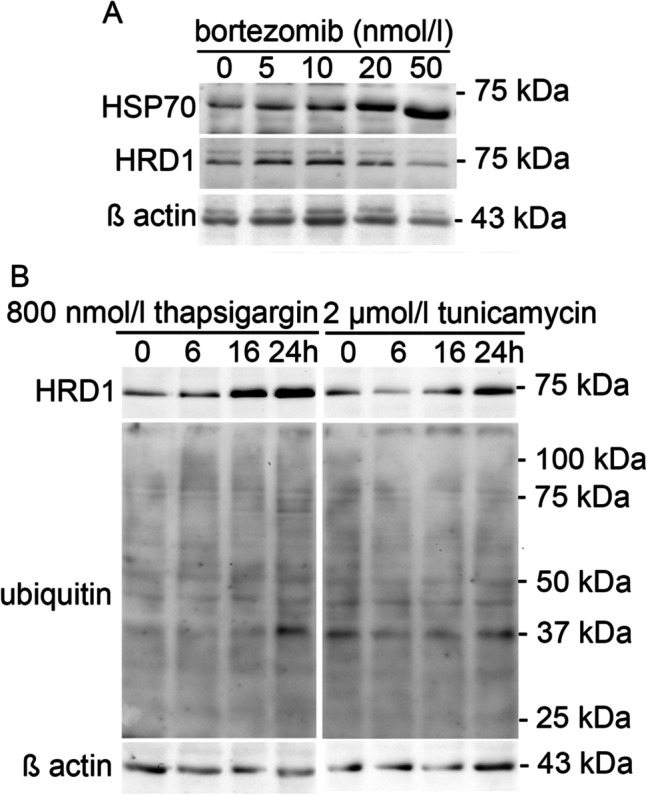
Impact of proteasome stress on expression of HRD1 and ER stress on accumulation of ubiquitin-conjugated proteins. A. Total cell extracts were prepared from SH-SY5Y cells after the treatment with indicated concentrations of bortezomib for 24 h. The effect of bortezomib on the levels of HRD1 and HSP70 was evaluated by Western blot analysis of total cell extracts as described in Materials and Methods. The representative blots are cropped from different parts of the same gel B. Total cell extracts were prepared from SH-SY5Y cells after the treatment with either thapsigargin at concentration 800 nmol/l or tunicamycin at concentration 2 µmol/l for 6, 16 and 24 h. The effect of ER stress on the levels of ubiquitin-conjugated proteins and HRD1 was evaluated by Western blot analysis of total cell extracts as described in Materials and Method. The representative blots are cropped from different parts of the same gel

Finally, using cellular models of proteasomal and ER stress, we have examined if there is a significant cross talk between proteasomal and ER stress. In order to check if the proteasomal stress can induce expression of HRD1, a specific molecular event of ER stress [42], we have performed a Western blot analysis of the SH-SY5Y cells treated with bortezomib that is a potent and specific inhibitor of 26 S proteasome. Based on our previous experiments, we have treated the cells with bortezomib at concentrations 5–50 nmol/l for 24 h since such treatment is associated with a significant accumulation of ubiquitin-conjugated proteins that is accompanied by increased expression of HSP70 [31, 32]. As shown in Fig. 6, inhibition of proteasome increases the expression of HSP70 but does not have an impact on HRD1 expression. We have also examined the impact of ER stress on the accumulation of ubiquitin-conjugated proteins, a typical molecular result of proteasomal stress. Based on our previous experiments, we have treated SH-SY5Y cells with both thapsigargin at a concentration of 800 nmol/l and tunicamycin at a concentration of 2 µmol/l for 6, 16 and 24 h since such treatments are associated with significantly increased expression of HRD1 [42]. After 16 and 24 h of treatment of SH-SY5Y cells with either thapsigargin or tunicamycin, we found increased expression of HRD1, but we did not observe accumulation of ubiquitin-conjugated proteins (Fig. 6).

## Discussion

The significant finding of our study is an association of global brain ischemia with an appearance of immunoreactivity of ubiquitin, PUMA and p53 in pyramidal neurons of the CA1 layer of the hippocampus 72 h after ischemic insults. These changes correlate with a delay and selectivity of neurodegeneration observed after global brain ischemia.

Degradation of aged and aberrant proteins via 26 S proteasome represents the most prominent function of UPS [47]. In addition, a wide range of important cellular functions that are dependent on the stability and intracellular localisation of proteins, protein-protein interactions, and transcriptional activity are controlled via mono- or poly-ubiquitinylation of specific proteins involved in the regulation of cell cycle, apoptosis, transcription and signal transduction [48]. Dysfunction or overload of UPS results in the accumulation of ubiquitin-conjugated proteins and has been implicated in the mechanisms of several pathophysiological processes, including ischemic neurodegeneration [29]. In vulnerable CA1 pyramidal neurons, significant accumulation of aggregates of ubiquitin-conjugated proteins has been observed mainly from the onset of reperfusion onward until delayed neuronal death occurs [24, 49–53]. Therefore, delayed neuronal death after transient global brain ischemia has also been attributed to the accumulation of ubiquitin-conjugated protein aggregates [28]. Accumulation of aggregates of ubiquitin-conjugated proteins observed after global brain ischemia was attributed to ischemia-induced inhibition of 26 S proteasome [25, 53, 54] and post-ischemic failure of autophagy [55]. In turn, aggregates of ubiquitin-conjugated proteins can inhibit 26 S proteasome [56] that can further contribute to their accumulation. Using Western blot analysis of total cell extracts from the hippocampus, we have detected significantly increased amounts of ubiquitin-conjugated proteins mainly 1 and 3 h after15 min of global brain ischemia [57, 58]. An increase of ubiquitin-conjugated proteins in the hippocampus during early reperfusion periods was associated with a significant decrease of free ubiquitin. The level of ubiquitin-conjugated proteins was still significantly elevated 24 and 72 h after ischemia, while the level of free ubiquitin was elevated back to the control values [58]. These results correlate with an appearance of ubiquitin immunoreactivity observed in CA1 pyramidal neurons 72 h after ischemia. In addition to the accumulation of ubiquitin-conjugated proteins, inhibition of proteasome is also associated with transcriptional and posttranscriptional events leading to increased expression of pro-apoptotic proteins PUMA and Noxa [32]. Although the involvement of PUMA and Noxa in neuronal cell death is still a matter of discussion, PUMA was suggested to be a key mediator of selective death of hippocampal CA1 neurons after proteasomal stress [26, 27]. In our experiments, we have also observed distinct immunoreactivity of both PUMA and p53 in pyramidal cells of the CA1 layer of the hippocampus 72 h after global brain ischemia. Expression of PUMA is controlled at the transcription level by p53 [38]. In turn, the stability of p53 is controlled by UPS [59]; thus, inhibition of UPS increases the level of p53 and, consequently, the expression of PUMA [27]. PUMA can bind to anti-apoptotic pro-survival proteins of the Bcl-2 family [38]. This process is associated with the initiation of mitochondrial apoptosis resulting in cell death. In the rat model of global brain ischemia, upregulation of PUMA in dying CA1 hippocampal pyramidal neurons was documented 5 days after ischemia in 10 min [60]. Increased expression of PUMA was also documented in CA1 cells 4 h after global cerebral ischemia [61] and in cortical neurons following focal cerebral ischemia [62, 63]. In the models of brain ischemia [61, 63] and cardiac arrest [64], the addition of Pifithrin-α, an inhibitor of p53, blocks the upregulation of PUMA, supporting the involvement of p53-dependent transcription of the *PUMA* gene after ischemia. In our previous study, we documented translocation of p53 to mitochondria isolated from rat hippocampus after 24 or 72 h following global brain ischemia in 15 min [65]. The translocation of p53 was not observed in mitochondria isolated from rat cortex after 1, 3, 24 or 72 h following global brain ischemia in 15 min. Finally, ischemic preconditioning prevented translocation of p53 to hippocampal mitochondria, which was associated with reduced degeneration of the cells in the CA1 layer of the hippocampus [65]. The involvement of p53 in neurodegeneration after global brain ischemia was further supported by recent studies [66, 67]. Upregulation of PUMA documented by both Western blot analysis and immunohistochemistry 4 h after global cerebral ischemia does not correlate with the delay of CA1 neuronal death at 72 h [23]. Our experiments have documented distinct PUMA immunoreactivity in CA1 pyramidal neurons 72 h after ischemia. Such a result correlates with the spatio-temporal distribution of degenerating cells as documented by Fluoro-Jade C staining. In addition to PUMA overexpression in CA1 neurons, an earlier study has documented the absence of expression of anti-apoptotic proteins Bcl-2 and Bcl-Xl in CA1 neurons after global brain ischemia [68]. In control brains, Bcl-2 immunoreactivity was not detected, and Bcl-Xl immunoreactivity was only present at a basal level. After 24 or 72 h following global brain ischemia in 15 min, immunoreactivity of both Bcl-2 and Bcl-Xl was noticeable at high levels in CA3 pyramidal neurons and a majority of DG granule cells but not in CA1 pyramidal neurons [68]. These results can further support the involvement of mitochondrial apoptosis in the mechanism of selective degeneration of neurons after global brain ischemia.

In addition to UPS dysfunction, increased expression of PUMA might be attributed to ER stress [69, 70]. ER stress was also considered as the key mechanism of neuronal death after global brain ischemia [20]. Several molecular events indicating induction of ER stress after global brain ischemia were documented, e.g. post-ischemic phosphorylation of eIF2α [71, 72], expression of CHOP [60, 73–75] and ATF4 [72, 75, 76] or splicing of XBP1 [74]. However, all these events were observed in an early period of reperfusion. An increase of ER resident chaperone BiP/GRP78 was documented by Western blot 3 days after global brain ischemia [75]. The same study has reported BiP/GRP78 immunoreactivity slightly elevated at 12 h and peaked at 1 day after ischemia in the CA1 layer, while BiP/GRP78 immunoreactivity was strong at 1 and 3 days after the ischemia in the CA3 layer. The overexpression of BiP/GRP78 after ER stress is generally considered as cytoprotective event. However, we did not observe significant changes in GRP78 expression after both naïve and preconditioned ischemia [57]. In contrast, immunoreactivity of CHOP, a transcription factor that drives expression of pro-apoptotic genes, was slightly increased at 1 day and peaked at 3 days after the ischemia in the hippocampal CA1 layer while CHOP immunoreactivity was strong at both 12 h and 1 day after the ischemia in the CA3 layer [75]. All described changes do not correlate with either delay or selectivity of neurodegeneration observed after global brain ischemia. Finally, ATF4 and CHOP immunoreactivity was documented in the CA1 layer 1 day after global brain ischemia [75]. It has to be noticed that both ATF4 and consequent CHOP expression could also be a result of the integrated stress response [77] that can also be triggered by proteasomal stress [78]. The idea about the possible activation of integrated stress response after brain ischemia was further strengthened by the recent study that has documented increased expression of CHPO 1 h after cardiac arrest [79]. In contrast, the expression of ER stress related proteins ATF6 and BiP/GRP78 was not altered [79]. The same study has documented the activation of caspase 3, which represents a specific molecular event associated with mitochondrial apoptosis. Concerning ER stress, we have observed the temporal disappearance of HRD1 immunoreactivity in pyramidal neurones of both CA1 and CA3 layers of the hippocampus but not in the cortex 24 h after global brain ischemia in 15 min. Some other molecular events that are characteristic features of UPR were not documented after global brain ischemia [80]. Since UPR is considered to be cytoprotective, it was concluded that ER stress is induced after global brain ischemia but the lack of protective responses results in the death of affected neurons [80, 81]. In agreement with such view, recent study documented neuroprotective impact of activation of ATF6 arm of UPR after cardiac arrest in mice [82]. The results presented in this study, as well as previously published results, do not correlate with both delay and selective degeneration of neurons observed after global brain ischemia, but are in accord with both in vivo and ex vivo experiments using ER stress inductor tunicamycin. Intracerebroventricular injection of tunicamycin into the mouse brain was associated with damage to the neuronal cells in either the CA3 layer [83] or the CA1 layer and DG [84]. Treatment of OHCs with tunicamycin at a concentration of 40 mg/ml for 24 h induced selective neuronal death in the DG without significant changes in the viability of the cells in the CA1 or CA3 area. Prolonged treatment for 48 h led to cell death in all areas of OHCs, while higher concentrations of tunicamycin led first to damage to CA1 and CA3 layers, and the cells of DG were later also affected [85]. Thus, tunicamycin treatment did not result in selective neurodegeneration like that observed after the inhibition of proteasome with epoxomicin.

Finally, ER stress is associated with activation of ERAD that depends on proteasomal degradation of aberrant proteins. Thus, ER stress can lead to UPS overload and consequent induction of proteasomal stress [35, 36]. Vice versa, UPS dysfunction could affect the process of ERAD that could result in accumulation of aberrant proteins in the ER lumen and consequent activation of ER stress responses. Using cellular models of proteasomal and ER stress, we have shown that there is no significant crosstalk between proteasome and ER stress at the level of typical molecular responses. Our results indicate that both proteasomal and ER stress represent two independent mechanisms of cellular responses to stress conditions that do not interact.

In conclusion, the results presented in this study are consistent with a view that UPS dysfunction and consequent p53-induced expression of PUMA result in mitochondrial apoptosis that represent the main mechanism responsible for selective and delayed degeneration of pyramidal neurons of the hippocampal CA1 layer in response to global brain ischemia. The question about selective dysfunction of UPS in pyramidal neurones of CA1 hippocampal layer remains to be open and requires further investigations.

## Data Availability

All data generated during and/or analysed during the current study are available from the corresponding author upon reasonable request.
